# The *in vivo *efficacy of two administration routes of a phage cocktail to reduce numbers of *Campylobacter coli *and *Campylobacter jejuni *in chickens

**DOI:** 10.1186/1471-2180-10-232

**Published:** 2010-09-01

**Authors:** Carla M Carvalho, Ben W Gannon, Deborah E Halfhide, Silvio B Santos, Christine M Hayes, John M Roe, Joana Azeredo

**Affiliations:** 1 IBB - Institute for Biotechnology and Bioengineering, Centre of Biological Engineering, University of Minho, Campus de Gualtar, 4710-057 Braga, Portugal; 2 University of Bristol, Department of Clinical Veterinary Science, Langford, North Somerset, BS40 5DU, UK

## Abstract

**Background:**

Poultry meat is one of the most important sources of human campylobacteriosis, an acute bacterial enteritis which is a major problem worldwide. *Campylobacter coli *and *Campylobacter jejuni *are the most common *Campylobacter *species associated with this disease. These pathogens live in the intestinal tract of most avian species and under commercial conditions they spread rapidly to infect a high proportion of the flock, which makes their treatment and prevention very difficult. Bacteriophages (phages) are naturally occurring predators of bacteria with high specificity and also the capacity to evolve to overcome bacterial resistance. Therefore phage therapy is a promising alternative to antibiotics in animal production. This study tested the efficacy of a phage cocktail composed of three phages for the control of poultry infected with *C. coli *and *C. jejuni*. Moreover, it evaluated the effectiveness of two routes of phage administration (by oral gavage and in feed) in order to provide additional information regarding their future use in a poultry unit.

**Results:**

The results indicate that experimental colonisation of chicks was successful and that the birds showed no signs of disease even at the highest dose of *Campylobacter *administered. The phage cocktail was able to reduce the titre of both *C. coli *and *C. jejuni *in faeces by approximately 2 log_10 _cfu/g when administered by oral gavage and in feed. This reduction persisted throughout the experimental period and neither pathogen regained their former numbers. The reduction in *Campylobacter *titre was achieved earlier (2 days post-phage administration) when the phage cocktail was incorporated in the birds' feed. *Campylobacter *strains resistant to phage infection were recovered from phage-treated chickens at a frequency of 13%. These resistant phenotypes did not exhibit a reduced ability to colonize the chicken guts and did not revert to sensitive types.

**Conclusions:**

Our findings provide further evidence of the efficacy of phage therapy for the control of *Campylobacter *in poultry. The broad host range of the novel phage cocktail enabled it to target both *C. jejuni *and *C. coli *strains. Moreover the reduction of *Campylobacter *by approximately 2 log_10_cfu/g, as occurred in our study, could lead to a 30-fold reduction in the incidence of campylobacteriosis associated with consumption of chicken meals (according to mathematical models). To our knowledge this is the first report of phage being administered in feed to *Campylobacter-*infected chicks and our results show that it lead to an earlier and more sustainable reduction of *Campylobacter *than administration by oral gavage. Therefore the present study is of extreme importance as it has shown that administering phages to poultry via the food could be successful on a commercial scale.

## Background

Worldwide, *Campylobacter *is recognized as the major etiologic agent in bacterial human diarrheoal disease [1-4]. Poultry, particularly chickens, account for the majority of human infections caused by *Campylobacter *[5,6]: *Campylobacter jejuni *and *Campylobacter coli *are the most prevalent species [2,7,8]. Surveys in Europe revealed that the prevalence of *Campylobacter*-positive poultry flocks varies from 18 to 90%, with the northernmost countries having substantially lower figures than southern European countries [9]. In the United States a survey indicated that nearly 90% of flocks were colonized [10]. The prevention of *Campylobacter *colonization has proven to be difficult [11] and therefore control of *Campylobacter *in poultry is an especially demanding goal to attain.

*Campylobacter *is commonly found in the gastrointestinal tract of poultry, where it replicates and colonises rapidly, even from very low inoculums [2,12]. When introduced into a flock, infection spreads rapidly by environmental contamination and coprophagy [9]. The problem of *Campylobacter *contamination of poultry is exacerbated following slaughter by cross-contamination from *Campylobacter*-positive to *Campylobacter*-negative carcasses during processing in the abattoir [13], showing that standard biosecurity measures on the processing plant are ineffective [14]. Even if it were possible to reduce the level of carcass contamination, such measures would be costly, difficult to maintain and restrictive. Consequently, another strategy is to operate control measures on the farm and thus significantly reduce colonization with *Campylobacter *prior to slaughter. As yet this has been difficult to achieve: strategies that successfully reduced *Salmonella *in broilers have proved to be only partially effective or totally ineffective in the control of *Campylobacter *colonization. These approaches include the treatment of feed with acid additives [15], vaccination of breeders [16,17] and competitive exclusion [18,19].

Due to increasing levels of antibiotic resistance in bacteria, the European Union has phased out the preventative use of antibiotics in food production [20]. Therefore, there is a pressing need to find alternatives to antibiotics that can be used to reduce the numbers of pathogens in animal products.

Bacteriophages are natural predators of bacteria, ubiquitous in the environment, self-limiting and self-replicating in their target bacterial cell [21]. Their high host-specificity and their capacity to evolve to overcome bacterial resistance [22] make them a promising alternative to antibiotics in animal production. There are several scientific studies on the use of phages to control animal diseases, namely those caused by *Salmonella *and *E. coli *[11,23-26]. *Campylobacter *phages have been isolated from several different sources such as sewage, pig and poultry manure, abattoir effluents, broiler chickens and retail poultry [27-35]. It has been demonstrated that they can survive on fresh and frozen retail poultry products [31]. Moreover they can exhibit a control effect on *Campylobacter *numbers, even in the absence of host growth, which is explained by the fact that some phages adsorb to the surface of the bacteria and just replicate when the metabolic activity of bacterium increases [36]. These make them potentially an important biocontrol agent of foodborne diseases.

The present study was undertaken to test the efficacy of a phage cocktail in reducing the levels of colonization by both *C. coli *and *C. jejuni *in broiler birds. In order to accomplish this task, experimental models of *Campylobacter *infection were designed and evaluated prior to the *in vivo *phage experiments. Moreover the best method of administering the phage cocktail was determined in order to ensure a high and consistent reduction in *Campylobacter *colonization. A further objective of this study was to evaluate the *in vivo *acquisition of phage resistance.

## Results

### Bacteriophage characterization

**Figure 1:**
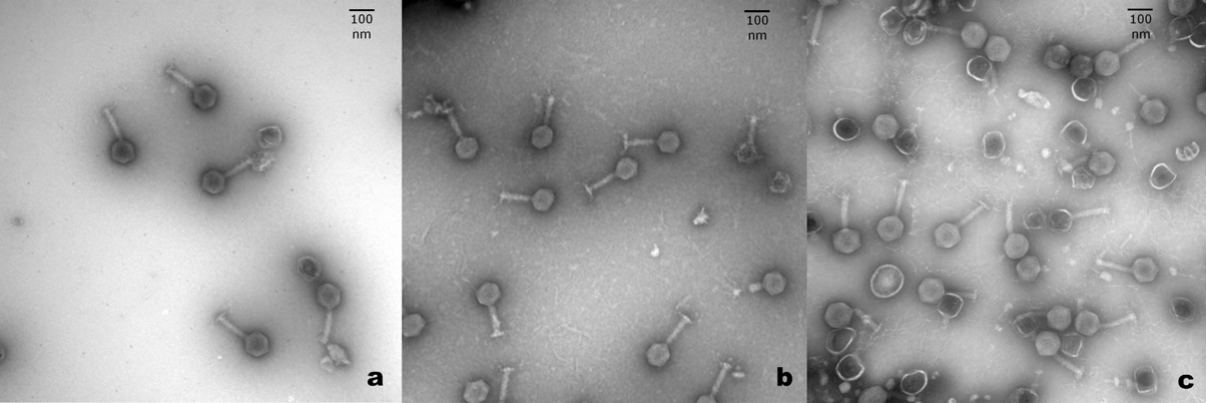
**Electron micrographs of the *Campylobacter *phages that composed the cocktail: (a) Phage phiCcoIBB12; (b) Phage phiCcoIBB35; (c) Phage phiCcoIBB37**. Phages were stained with 1% uranyl acetate and observed with a transmission electron microscopy. There was no difference in morphology between the three phages. They have an icosahedral head of approximately 100 nm in diameter and a contractile tail with 140 × 17 nm average length. This morphology is typical of the members of the *Myoviridae *family.

**Figure 2:**
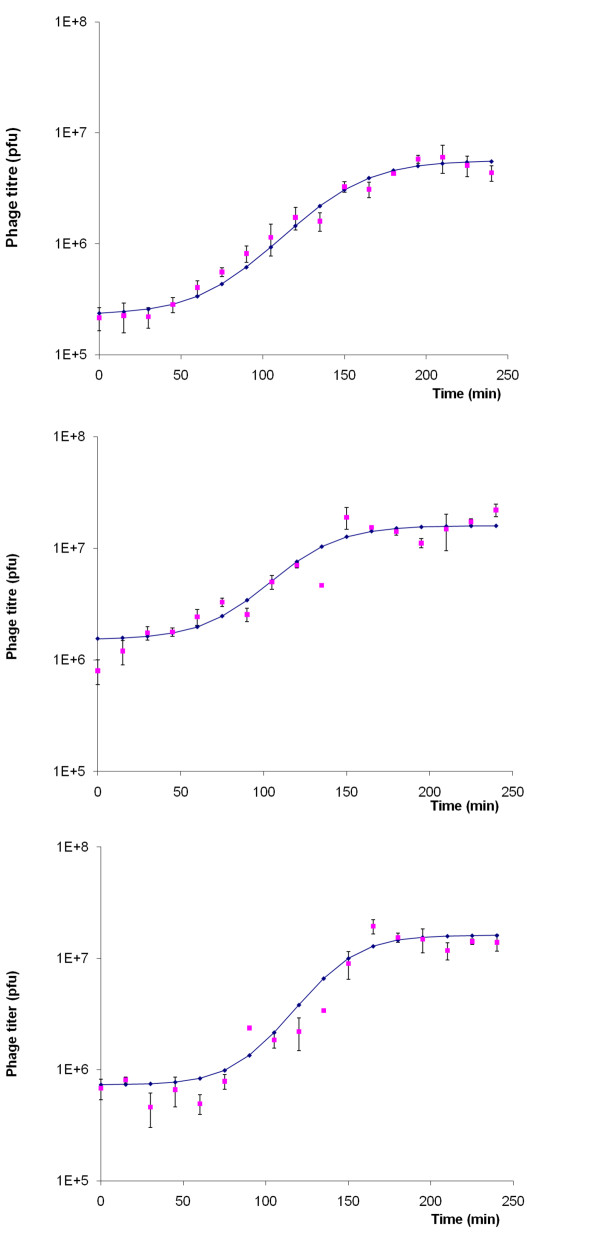
**Single-step growth curve of the *Campylobacter *phages that composed the cocktail: (a) Phage phiCcoIBB35; (b) Phage phiCcoIBB37; (c) Phage phiCcoIBB12**. Single-step growth experiments were performed in order to assess the latent period and burst size of a single round of phage replication: phage phiCcoIBB35 has a burst size of 24 pfu and a latent period of 52.5 min; phage phiCcoIBB37 has a burst size of 9 pfu and a latent period of 67.5 min; phage phiCcoIBB12 has a burst size of 22 pfu and a latent period of 82.5 min. Samples were taken every 15 min for 4 h. The data was fitted to a four-parameter symmetric sigmoid model. Non-linear regression was performed to calculate the latent period and burst size. Error bars represent the standard deviation.

The phage cocktail used in the present study was composed of three phages (phiCcoIBB35, phiCcoIBB37, phiCcoIBB12) previously isolated from poultry intestinal contents and selected on the basis of their broad lytic spectra against food and clinical *C. coli *and *C. jejuni *strains [35]. The three phages showed different and complementary lytic spectra [35]. They were morphologically, genetically and physiologically characterized by transmission electron microscopy (TEM), pulsed field gel electrophoresis (PFGE), restriction fragment length polymorphism (RFLP) and single-step growth experiments. Morphologically the three phages have a similar structure and size, each possessing an icosahedral head (average diameter of 100 nm) and a contractile tail (140 × 17 nm average length) with tail fibres at the distal end. These morphologies are typical of the *Myoviridae *family of lytic phages [37]. Electron micrographs are presented in Figure 1. PFGE and RFLP experiments showed each of the three phages to have a genomic DNA size of approximately 200 kb that was not cut by any of the restriction enzymes tested. Single-step growth curves results (Figure 2) showed that the burst size of phage phiCcoIBB35 was 24 pfu with a latent period of 52.5 min; the burst size of phage phiCcoIBB37 was 9 pfu with a latent period of 67.5 min and the burst size of phage phiCcoIBB12 was 22 pfu with a latent period of 82.5 min.

### Animal experiments

#### *Campylobacter *colonization models

**Figure 3:**
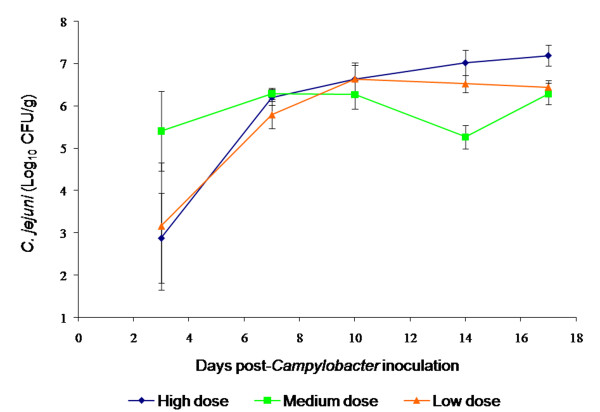
**Colonization of chicks by *Campylobacter jejuni *2140CD1 after challenge with a range of dose levels**. Eighteen, one day-old chicks were randomly assigned to one of three groups receiving by oral gavage different concentrations of 0.1 ml of PBS *C. jejuni *2140CD**1:**low dose (7.5 × 10^4^cfu); medium dose (1.0 × 10^6^cfu) and high dose (5.5 × 10^7^cfu). Faecal samples were collected from all birds at intervals and *Campylobacter *and phages enumerated. Error bars represent the standard error of the mean.

Prior to testing the phage efficacy *in vivo *it was necessary to determine the optimum dose of *Campylobacter *needed to produce consistent *Campylobacter *levels in faeces. The essential parameters of the infection model were therefore set to mimic natural *Campylobacter *colonisation: the colonisation level to be between 1 × 10^6 ^and 1 × 10^9^cfu/g of faeces, the number found in commercial broiler flocks [38], and the birds should be asymptomatic. The *C. jejuni *2140CD1 numbers presented in Figure 3 show that the geometric mean colonisation level at three days post-infection (dpi) was lower than at subsequent sampling points. The logarithmic mean colonisation levels, excluding 3dpi, were 2.2, 1.1, and 5.8 × 10^6^cfu/g for the low, medium and high dose groups respectively and the standard error of the mean was approximately 0.3 cfu/g. The primary reason for the lower mean in the 3dpi sample point was that within each group some of the samples were negative for *C. jejuni *2140CD1, which reduced the mean levels: four out of seven birds in the low dose group, one out of seven birds in the medium dose group and three out of seven birds in the high dose group were negative. These negative samples were represented by birds that were not colonized or birds which the *Campylobacter *numbers in faecal samples was inferior to the detection limit (500 cfu/g). Similar experiments were performed to establish the colonization model for the *C. coli *strain used in this study (*C. coli *A11) and a consistent number of 1.7 × 10^6^cfu/g bacterial cells was found in the faeces of the birds after 7dpi.

#### Phage cocktail administration

Prior to the phage cocktail administration experiments, all birds were screened for phages active against the inoculum *Campylobacter *and proved to be negative.

In a preliminary experiment (data not shown), the phage cocktail was administrated by oral gavage to one-week old chicks infected with *C. jejuni *2140CD1. The faecal samples collected at all sample time points presented *Campylobacter *but did not contain any of the phages administered. This suggested that the phages might have been sensitive to low pH such as occurs during passage through the proventriculus and gizzard. The use of an antacid has been demonstrated to improve the ability of phages to survive low acidity in the digestive system [39] and therefore in the following trials (Experiment 1 and Experiment 2) the phage cocktail was administered with CaCO_3_.

In Experiments 1 and 2 the results show that the numbers of *Campylobacter *in the control group were stable throughout the experiments (no statistically significant difference), which shows that the birds were well colonized. Moreover the fact that the treated groups and the untreated groups had the same level of *Campylobacter *colonization at the beginning of the experiments ensures that accurate comparisons between these two groups can be made.

In Experiment 1, the phage cocktail was administered by oral gavage to one-week old chicks infected with *C. jejuni *2140CD1. In order to determine the best phage delivery policy, in Experiment 2 a comparison was made of administering the phage cocktail by oral gavage and by incorporating it into the chicks' food, using chicks infected with *C. coli *A11.

**Figure 4:**
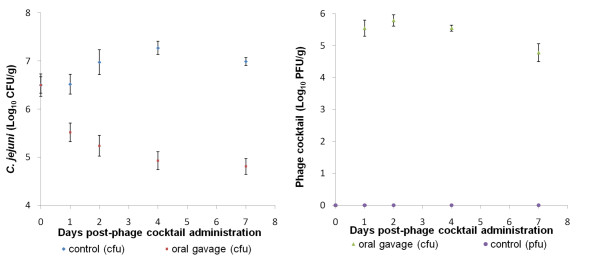
**Numbers of *Campylobacter jejuni *2140CD1 (a) and phages (b) in faeces from broilers orally administered a phage cocktail by gavage**. Thirty day-old chicks were inoculated with *Campylobacter jejuni *2140CD1. One week later the birds were randomly assigned to a treated group or an untreated group and were inoculated by oral gavage with antacid containing 1 × 10^6^pfu of a phage cocktail, or antacid only respectively. Faecal samples were collected from all birds at intervals and *Campylobacter *and phages enumerated. Error bars represent the standard error of the mean. At 2 dpa, 4 dpa and 7 dpa there is a significant difference between control and infected group at P < 0.05.

**Figure 5:**
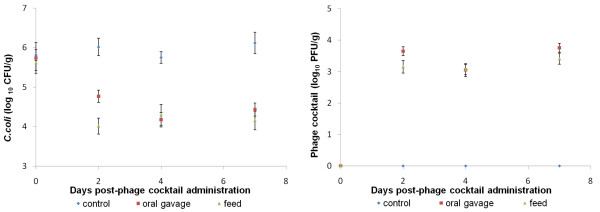
***Numbers of Campylobacter coli *A11 *(a) *and phages (b) in faeces from broilers orally administered phage by food or by oral gavage**. Forty-five, day-old chicks were inoculated with *Campylobacter coli *A11. One week later the birds were randomly assigned to one of three groups, a non-treated group and two treated groups: a group receiving the phage cocktail by oral gavage; and a group receiving the phage cocktail in feed. Birds were inoculated with antacid only, antacid containing 1 × 10^6^pfu phage cocktail or antacid followed by feeding with the phage cocktail laced with 1.5 × 10^7^pfu, respectively. Faecal samples were collected from all birds at intervals and *Campylobacter *and phages enumerated. Error bars represent the standard error of the mean. At 1 dpa, 2 dpa, 4 dpa and 7 dpa there is a significant difference between control and infected groups at P < 0.05.

For Experiments 1 and 2, the data show a reduction in the number of *Campylobacter *in the chicks that received the phage cocktail when compared to the chicks from the untreated group (control group) which received only antacid (Figures 4 and 5 respectively). The log_10_cfu/g difference between these groups is presented in Table 1. After phage administration, the colonization values from the chicks belonging to the treated groups were lower than the values from the chicks that received no treatment (control group). In fact, using one-way ANOVA, it can be said that each value of *Campylobacter *counts from the treated and the control group was statistically significant different (P < 0.05) during the experimental period. In Experiment 1, at four days post-phage administration (4 dpa) it was already possible to see a reduction of 2.34 log_10 _cfu/g in the numbers of *C. jejuni *2140CD1 when comparing the untreated and treated groups. This reduction was consistent through the experiment and at 7 dpa it was 2.18 log_10_cfu/g. In Experiment 2 the results show that phage cocktail delivered by food was effective and resulted in a slightly higher reduction (approximately 2 log_10 _cfu/g) in pathogen numbers than the phage cocktail administered by oral gavage (1.7 log_10 _cfu/g reduction), when compared to the untreated group at the end of the experimental period (7 dpa). However a reduction of 2 log_10 _cfu/g in *Campylobacter *numbers in faeces was already observed at 2 dpa when the phage cocktail was given by food, while at this time point the reduction was only 1.25 log_10 _cfu/g in the faecal samples of the group that received the phage cocktail by oral gavage. We believe that this trial was not compromised by the pecking order of the chickens because the birds were observed during the trial in order to assure that all of them had eaten. Moreover the low value of the standard error (0.2 pfu/g) of the phage titer after two days of treatment demonstrated that there were small variations in the dose of phage that each bird received.

**Table 1:** Difference between the geometric means of the *Campylobacter *titre from broilers with and without the phage cocktail administration

Experiment	Administration route	*Campylobacter *titre (log_10_cfu/g)		
		
		Day 2	Day 4	Day 7
Experiment 1	Oral Gavage	1.74	2.34	2.18
Experiment 2	Oral Gavage	1.25	1.58	1.69
	Feed	2.00	1.45	1.96

The phage titers from faecal samples of the chicks infected with *C. jejuni *and *C. coli *were log_10 _5.3 pfu/g and log_10 _3.4 pfu/g for Experiment 1 and Experiment 2 respectively. These values remained approximately constant throughout the experimental period showing that phages delivered to chicks (either by oral gavage or in feed) were able to replicate and therefore able to reduce the *Campylobacter *populations.

Previous studies [40,41] have used the number of *Campylobacter *in the caecal contents of the birds as a measure of *Campylobacter *colonisation levels in the GI tract of chickens [41,34]. Although this may be a representative of colonisation levels, the animals must be killed and dissected to obtain the sample. This can lead to the use of an excessive number of birds when multiple time points are required to evaluate phage levels over the lifetime of the bird. Therefore in the present study cloacal swabs were used to determine colonisation levels as they can provide a rough estimate of the numbers of bacteria in the cecum of chickens [42]. Moreover these samples show the kinetics of colonization as multiple samples can be taken from single birds. Another advantage is that it represents the number of *Campylobacter *being released from the bird into the environment and so directly correlates to the capacity of the bird to transmit the bacteria.

#### *In vivo *acquisition of phage resistance

In order to evaluate the acquisition of resistance to the phage cocktail in *Campylobacter jejuni *infected and treated birds, a total of 300 *Campylobacter *colonies, isolated from each infected bird belonging to the treated group in Experiment 1, were checked for their sensitivity to the phage cocktail, before and after phage administration. We observed that before phage treatment, 6% of the isolated colonies were resistant to the phage and at 7 dpa 13% of the isolated colonies were phage resistant. Although the results from these experiments are not easily interpreted because bacteria that had not been exposed to phage already demonstrated a certain degree of phage resistance, the key conclusion is that the resistant phenotype could have been selected for during therapy. If that was the case, then the resistant phenotype would soon become the dominant phenotype after therapy began. This may be connected to previous observations that resistant bacteria lose fitness and are out-competed by the non-resistant phenotype in the intestines, despite being sensitive to the phage that is present [40]. To test this hypothesis seven groups of 15 birds were inoculated with phage-sensitive and phage-resistant *Campylobacter *strains re-isolated from birds used in the previous trial. The numbers of *Campylobacter *in faeces from each bird was enumerated at seven days post-inoculation (Table 2). There was no significant difference between any of the groups (P > 0.05 by *t*-test). This suggests that the resistant phenotype was not hindering the ability of the *Campylobacter *to colonise the chickens. However it may have been the case that *in vivo *the resistant phenotype was rapidly lost so no lack of fitness was evident. In order to test this hypothesis we randomly selected three *Campylobacter *colonies from faecal samples from each infected chicken of each of the groups and determined their sensitivity to the phage cocktail (Table 2). Interestingly, 86.2% of the colonies isolated from chickens infected with resistant strains isolated before phage treatment lost their resistant phenotype and 54% of the resistant strains isolated in phage treated chickens reverted their resistant phenotype to a sensitive one. These results are not in accordance with Loc Carrillo *et al*. [40] in which 97% of resistant phenotype reverted back to phage sensitive strains.

**Table 2:** Geometric means of *Campylobacter *titre (log_10_cfu/g) in faeces of broilers after 7 dpi with phage sensitive and phage resistant *Campylobacter *strains; (%) of resistant *Campylobacter *strains to the phage cocktail

*Campylobacter *phage sensitivity	*Campylobacter *titre (log_10_cfu/g)	Resistant strains (%)
Sensitive	6.55	nd
Resistant (a)*	6.50	13.8
Resistant (b)*	6.29	46

*(a) Strains isolated from non-phage treated chickens and (b) Strains isolated from phage treated chickens

## Discussion

The characterization of the three *Campylobacter *phages that compose the cocktail is in accordance with the majority of *Campylobacter *phages reported in the literature [29,31,34,40,43,44]. The only restriction enzyme that has been used successfully to digest the DNA of some *Campylobacter *phages is *Hha*I, but even this enzyme did not yield results for the phages used in the present study. Possible explanations for these results include: the phage genomes may have lost restriction sites due to selective pressures from restriction modification systems; the phage genomes may have encoded nucleotide-modifying enzymes such as methyltransferases that would have modified the bases at the restriction sites; the phage genomes may contain unusual bases. Further studies such as phage genome sequencing would be needed in order to understand the refractory nature of the DNA of the *Campylobacter *phages.

To our knowledge there is just one report in the literature where the burst size and latent period parameters were calculated for *Campylobacter *phages, i.e. 1.957 virions per cell and 1.312 h respectively [45]. The phages phiCcoIBB35, phiCcoIBB37 and phiCcoIBB12 that were used in the present study have smaller latent periods (52.5 min, 67.5 min and 82.5 min) and higher burst sizes (24, 9 and 22 virions per cell) respectively.

In order to evaluate the efficacy of the three phages in the *in vivo *trials, it was necessary to recreate experimentally *Campylobacter *colonization in chicks. The model used revealed a successful colonisation; no birds in any of the groups showed any overt symptoms of disease, colonisation or stress even at the highest dose of *Campylobacter *administered. This asymptomatic carriage mimics *Campylobacter *colonisation in commercial flocks. The dose of *Campylobacter *appeared to have little effect on the outcome of subsequent colonisation levels. The logarithmic mean level of colonisation of the three groups was 2.4 × 10^6^cfu/g, which is within the range of the infection levels found in commercial broiler flocks: 1 × 10^6 ^to 1 × 10^9^cfu/g [38] and hence is an appropriate level for the experimental model. The data shows that *Campylobacter *had not consistently colonised all the birds by 3dpi. Although the reasons for *Campylobacter *colonization failure of young birds are still unclear, these negative colonized chickens may have maternal antibodies which protects them from *Campylobacter *colonization [46]. In all subsequent time points all birds were colonised. This suggests that if a trial is to evaluate the reduction of *Campylobacter *levels from colonised birds, it is essential to allow time for *Campylobacter *to become established in the gut of the chicks before phage treatment is initiated. Therefore, in the present study phage treatment was performed after seven days post-infection.

The results of the *in vivo *trials show that the phage cocktail was able to reduce the number of *C. jejuni *(Experiment 1) and *C. coli *(Experiment 2) colonisation in chickens, by approximately 2 log_10 _cfu/g. Moreover this reduction persisted throughout the experimental period. Other studies [40,41] produced a similar reduction of *Campylobacter *counts at the end of the experimental period. However that reduction was of transient nature in comparison to our study, where a sustained reduction in *Campylobacter *numbers was obtained during the seven days trial. A phage therapy that produces this kind of reduction of a pathogen would probably allow the phage administration to the birds at any point in the production cycle. The advantages of giving the phage early in production would be that environmental contamination would be minimised and that only a proportion of the flock would need treating as the phage would be spread naturally in the environment to all birds. However this strategy does carry a risk of resistance emerging and reducing the efficacy of treatment. In fact, *Campylobacter *strains resistant to phage infection were recovered from phage-treated chickens at a frequency of 13%. However resistance to the phage cocktail was found in *Campylobacter *in chickens before phage therapy, which means that bacteria can naturally acquire phage resistance. Nevertheless, following phage treatment an increase in the resistant population was observed meaning that phages might have selected for resistant strains. In our results and conversely to results described by Loc Carrillo *et al*. [40] the resistant phenotype did not lose the ability to colonise the chicken gut and did not completely revert to sensitive type. This can be pointed out as a major drawback of phage therapy. So, in order to overcome this problem the best strategy of phage administration is a short time before slaughter. Additionally, it is recommended that when selecting the phages that will compose the cocktail an additional criterion should be the ability to infect other phage resistant *Campylobacter *phenotypes.

In the present study, two phage administration strategies were assessed: oral gavage and food incorporation. Oral gavage permitted the delivery of accurate doses directly to the gastro-intestinal (GI) tract of individual birds. However if phage therapy is to be utilised by the poultry industry then the phage product must be simple and cheap to administer to flocks consisting of several thousand birds. We demonstrated that application of phage therapy can be successfully achieved in food leading to a reduction similar to that achieved by oral gavage. Moreover this reduction was earlier in comparison to the group that received the phage cocktail by oral gavage which can be explained by the protective effect of food that hampers the low pH from inactivating the phages [47]. These results are of extreme importance as this route of phage administration can provide a viable strategy for delivery of phage in a commercial context. Phages could also be given in the drinking water, however preliminary experiments showed that phage needed to be administrated with antacid and this could prove more difficult to deliver with the water than as an inclusion in the feed.

Moreover, in our study the phage cocktail was administered as a single dose to *Campylobacter-*infected chicks 7dpi. A single dose of phage is, in comparison to multiple doses [41], an easier and more feasible strategy in a farm situation.

It must be noted that the present model does not comprise all the variables that can play a role in the use of phages to control *Campylobacter *in poultry. Firstly, this model considers the use of phages as a therapy and not as a prophylactic measure. Secondly, in the present work birds were challenged with *Campylobacter *at one-year-old, but in a real commercial context birds just get colonized with *Campylobacter *after two weeks of age. However, these conditions were not tested in our experiments as it is very difficult to maintain chicks free of pathogens. An additional limitation of the model was the limited time course of the experiments (seven days). Nevertheless, the model described herein is a proof of principle that *Campylobacter *phages given orally or administered in feed can effectively reduce the *Campylobacter *colonization levels. Further studies need to be undertaken in order to test phage effectiveness in older chickens, their use as prophylactic agents and longer time course trials in order to reflect the production cycle.

## Conclusions

The phage cocktail was able to reduce *C. coli *and *C. jejuni *in infected poultry by approximately 2 log_10_cfu/g, which is of great importance as they are the most prevalent *Campylobacter *species found in positive *Campylobacter *flocks. Moreover mathematical models indicate that a 2 log_10_cfu/g reduction of *Campylobacter *on the chicken carcasses could lead to a 30-fold reduction in the incidence of campylobacteriosis associated with consumption of chicken meals [48]. The phage cocktail administered in feed led to an earlier reduction in *Campylobacter *titre than when given by oral gavage and thus this method can be easily and successfully used under commercial condition in a poultry unit. Another important aspect of the present study is that as the phages that composed the cocktail were isolated from poultry carcasses, their use to reduce *Campylobacter *colonisation in the live birds would not introduce any new biological entity into the food chain.

## Methods

### Bacterial strains

For the single-step growth experiments, two wild type strains of *C. coli*, isolated from poultry and poultry products, were used as the hosts of the three phages that composed the cocktail (*C. coli *A11, host of phages phiCcoIBB35 and phiCcoIBB37; *C. coli *8907, host of phage phiCcoIBB12). For the animal trials, two *Campylobacter *strains were chosen: *C. coli *A11 and *C. jejuni *2140CD1 (isolated from chickens in a commercial production unit).

### Bacteriophage characterization

For the phage cocktail, three phages (phiCcoIBB35, phiCcoIBB37, phiCcoIBB12) were selected from a panel of 43 phages, isolated from poultry carcasses, based on their broad lytic spectra against *C. coli *and *C. jejuni *strains [35]. These phages were characterized by transmission electron microscopy (TEM), pulsed field gel electrophoresis (PFGE), restriction fragment length polymorphism (RFLP) and single-step-growth experiments.

#### TEM characterization

PEG-purified phage samples were applied for 1 min on glow-discharged 400-mesh Formvar Carbon copper grids (Ted Pella) and blot dried. The grids were stained with 1% uranyl acetate for 1 min. The samples were observed under a JEOL transmission electron microscope at 60 kV and images recorded (Figure 1).

#### PFGE

Phage DNA was extracted using the SDS-proteinase K protocol described by Sambrook and Russell [49] for lambda phage. The PFGE determination was performed as described by Lingohr and Johnson [50].

#### Restriction Profile

Restriction endonuclease digests was performed using the following enzymes: *Hha*I, *Eco*RV, *Eco*RI, *Xba*I, *Hin*dIII, *Dde*I in accordance to the manufacturer's instructions i.e. 1 h at 37°C (Fermentas Life Sciences). Electrophoresis of the digested DNA was performed at 90 V for 2 h using 1.5% agarose Tris-acetate-EDTA gel.

#### Burst size and Latent Period (Single-step growth curve)

Single-step growth experiments were performed in order to assess the latent period and burst size of a single round of phage replication. Briefly, host cells were grown to early exponential phase (OD_600 nm _= 0.3) in 100 ml of NZCYM broth (Sigma Aldrich, Poole, UK) and incubated with shaking at 42°C in a microaerobic atmosphere (5% O_2_, 5% H_2_, 10% CO_2_, 80% N_2_). They were then infected with the particular phage at a multiplicity of infection (MOI) of 0.001. Samples were taken every 15 min for 4 h and the titre determined immediately by the double-layer agar plate method in NZCYM agar (NZCYM broth with 1% agar (Sigma Aldrich). Three independent replicates of each single-step growth experiment were performed. The mean values obtained from these experiments are presented on Figure 2. The data were fitted to a four-parameter symmetric sigmoid model. Non-linear regression was performed to calculate the latent period and burst size.

### Animal experiments

The animal experiments were designed to obtain sufficient high quality data to achieve objectives whilst conserving available resources including animals, money, work hours and consumables. Therefore all animal experiments were carried out according to the UK Animals (Scientific procedures) Act 1986 (licence number PPL 30/2322), which stipulates that any experiments on live animals requires a justification of numbers used to ensure that meaningful data is gathered from the least number of animals.

One-day-old Ross broiler chicks (Faccenda, Brackley, UK) were obtained from a commercial hatchery and were housed in a controlled environment in floor boxes under strict biosecurity. Swabs of faecal samples were collected from each individual bird prior to the experiment starting to ensure the absence of any *Campylobacter *and any phages against the *Campylobacter *strains which were used for infection. Faecal samples were then pooled in groups of six and 1 g inoculated into 10 ml of Bolton broth (Oxoid, Basingstoke, UK) supplemented with cefaperazone, vancomycin, trimethoprim and cycloheximide (Oxoid) and 5% lysed horse blood (Oxoid). The broths were incubated at 42°C in a microaerobic atmosphere overnight and then plated onto mCCDA (Oxoid) and incubated in the same manner for 48 h. Plates were then checked for growth of *Campylobacter*. The screen for phages was performed using the 'phage detection using semi-solid agar' methodology detailed below.

#### Colonization model

Three groups of six birds, designated low, medium and high dose were used: each group received a crop gavage of 0.1 ml of PBS (Sigma) containing respectively 7.5 × 10^4^, 1.0 × 10^6^, or 5.5 × 10^7^cfu of an overnight culture (42°C in microaerobic atmosphere) of *C. jejuni *strain 2140CD1. Swabs of faecal samples were collected from each individual bird at 3, 7, 10, 14, and 17 dpi (days post-infection). *Campylobacter *enumeration was performed by serial ten-fold dilutions in SM buffer (0.05 mol/l Tris-HCl [pH 7.5], 0.1 mol/l NaCl, 0.008 mol/l MgSO_4_) followed by plate counts on mCCDA plates (Oxoid). The same experiments were performed with the *C. coli *A11, with the exception that only the medium dose of inocula (1.0 × 10^6^cfu) was used to infect the chicks.

#### Phage cocktail administration

Two animal experiments were conducted. In Experiment 1, thirty one-day-old chicks were inoculated with 1 × 10^6^cfu of *C. jejuni *2140CD1 in 0.1 ml PBS by oral gavage and housed together for seven days. One week later faecal samples were collected to screen for phage active against the *Campylobacter *strain in the inocula using the 'phage detection using semi-solid agar' methodology detailed below. The chicks were then randomly divided into groups of 15 and inoculated with 1 × 10^6^pfu of the phage cocktail in 1 ml of antacid (30% CaCO_3_), or given antacid only (control group). In Experiment 2, *C. jejuni *2140CD1 was substituted for *C. coli *A11 and two methods of phage administration were compared: oral gavage and in food. The administration in feed was achieved by withdrawing the normal feed for 3 h and then dosing the chicks with 1 ml of antacid. The group of chicks were then given 45 g of chick crumbs laced with 1.5 × 10^7^pfu phage cocktail in 1.5 ml of SM buffer. After all of the food had been consumed (~1 h) normal feed was re-introduced. Birds were observed during this feeding period to ensure they had all fed. Swabs of faecal samples were collected from each individual bird at intervals after the phage cocktail had been administered and *Campylobacter *and phages enumerated. The samples were weighed and nine volumes of SM buffer added to produce a 1/10 faecal suspension (minimum of 1.5 ml of SM buffer was added).

#### *Campylobacter *enumeration

A ten-fold dilution series in 10 mM MgSO_4 _was prepared from each faecal sample collected and 20 μl aliquots of each dilution were spread on half plates of mCCDA agar (Oxoid). The plates were incubated at 42°C in a microaerobic atmosphere for 48 h and characteristic *Campylobacter *colonies were counted to determine the titre in the original faecal sample.

#### Phage detection using semi-solid agar

Cultures of *C. jejuni *2140CD1 or *C. coli *A11 were streaked on 5% horse blood agar (Oxoid) and incubated overnight at 42°C in a microaerobic atmosphere. The bacteria were harvested into 1.5 ml of 10 mM MgSO_4_, and added to 50 ml of molten (55°C) 'top agar': NZCYM broth (BD Biosciences, Oxford, UK) with 0.7% Agar (BD Biosciences).

For screening the pooled faecal samples, a semi-solid overlay method was used: the molten agar and the target *Campylobacter *strain suspension (approximately 5 ml) was poured onto an NZCYM plate and allowed to set. The pooled faecal samples were treated with 20% (w/v) chloroform, vortexed and then centrifuged at 8600 g for 5 min. Each supernatant was then applied to the over-layered plates in a 20 μl drop. Plates were then incubated at 42°C in a microaerobic atmosphere. For enumeration of phage, a ten-fold dilution series was prepared from each treated sample and a 20 μl aliquot placed in (the centre of) one well of a 6-well tissue culture plate. Three ml of the suspension of *Campylobacter *and molten agar was then added to each well, gently mixed and then the plates were incubated at 42°C in a microaerobic atmosphere overnight. Plaques in the bacterial lawn were counted after incubation and the phage titre determined.

#### *In vivo *acquisition of phage resistance

Swabs of faecal samples were collected from birds colonized with *Campylobacter jejuni *strain 2140CD1 at 0 dpa and at 7 dpa in Experiment 1. A ten-fold dilution series in 10 mM MgSO_4 _was prepared from each faecal sample collected and 20 μl aliquots of each dilution were spread on half plates of mCCDA agar (Oxoid). The plates were incubated at 42°C in a microaerobic atmosphere for 48 h and ten characteristic *Campylobacter *colonies were randomly selected from each faecal sample and their sensitivity to the phage cocktail was tested. Briefly, a drop of the phage cocktail (10 μ) was added to lawns [35] of each colony pick and the plates incubated overnight at 42°C in microaerobic atmosphere. The appearance of clear zones around the point of application was recorded as the ability to lyse that strain.

Seven groups of 15 birds were inoculated with 0.1 m of PBS containing 1.0 × 10^6^cfu of an overnight culture (42°C in microaerobic atmosphere) of the *Campylobacter jejuni *strains re-isolated from birds used in the previous trial: two groups received one of each of two separate sensitive *Campylobacter *strains, three groups received the *Campylobacter *resistant strains isolated from treated birds and finally two groups received the resistant *Campylobacter *isolated from birds before phage treatment. The numbers of *Campylobacter *in faeces from each bird was enumerated at seven days post-inoculation. Swabs of faecal samples were collected from the infected birds and three *Campylobacter *colonies isolates were selected at random from each faecal sample and checked for their sensitivity to the phage cocktail, as previously described.

#### Statistical treatment of data

Statistical differences in faecal samples between control and the phage cocktail treatment groups, between the phage cocktail treatment groups themselves and between the sampling points within each group were assessed by using the one-way ANOVA test.

## Authors' contributions

CC and BG designed and planned the experiments, analyzed the data and wrote the manuscript. CC, BG, CH and DH performed the animal trials experiments. CC and SS performed the phage characterization experiments. CC, BG and SS made the statistical analysis of the data. JA and JR supervised and participated in the conception of the study, contributed with materials and reagents and revised the manuscript. All authors read and approved the final manuscript.
